# Supplementary material for: Two-Photon Absorption and Multiphoton Excited Fluorescence of Acetamide-Chalcone Derivatives: The Role of Dimethylamine Group on the Nonlinear Optical and Photophysical Properties
Source: Molecules. 2023 Feb 6;28(4):1572. doi: 10.3390/molecules28041572 (PMC9963839; doi:10.3390/molecules28041572)
Supplement: Supplementary file 1 [file molecules-28-01572-s001.zip › molecules-2076507-supplementary.pdf]

Two-photon absorption and multiphoton excited fluorescence of acetamide-chalcone derivatives: the role of dimethylamine group on the nonlinear optical and photophysical properties

André Gasparotto Pelosi[1], Eli Silveira-Alves Jr[2], Leandro Henrique Zucolotto Cocca[1], João Victor Valverde[1], Guilherme Roberto Oliveira[2], Daniel Luiz da Silva[3], Leonardo De Boni[1], Pablo José Gonçalves[2,4] and Cleber Renato Mendonca[1]

1 Instituto de Física de São Carlos - Universidade de São Paulo
2 Instituto de Química – Universidade Federal de Goiás
3 Departamento de Ciências da Natureza, Matemática e Educação – Universidade Federal de São Carlos (Araras)
4 Instituto de Física - Universidade Federal de Goiás
Author to whom correspondence should be addressed: crmendon@ifsc.usp.br

Supporting Information

1) Compounds

The studied compounds were in powder form and were solubilized in DMSO solution. Compounds ChN(CH3)2 and ChNO2 presented yellow coloration in solution and the other compounds presented a translucid appearance.

2) Molar absorption coefficient

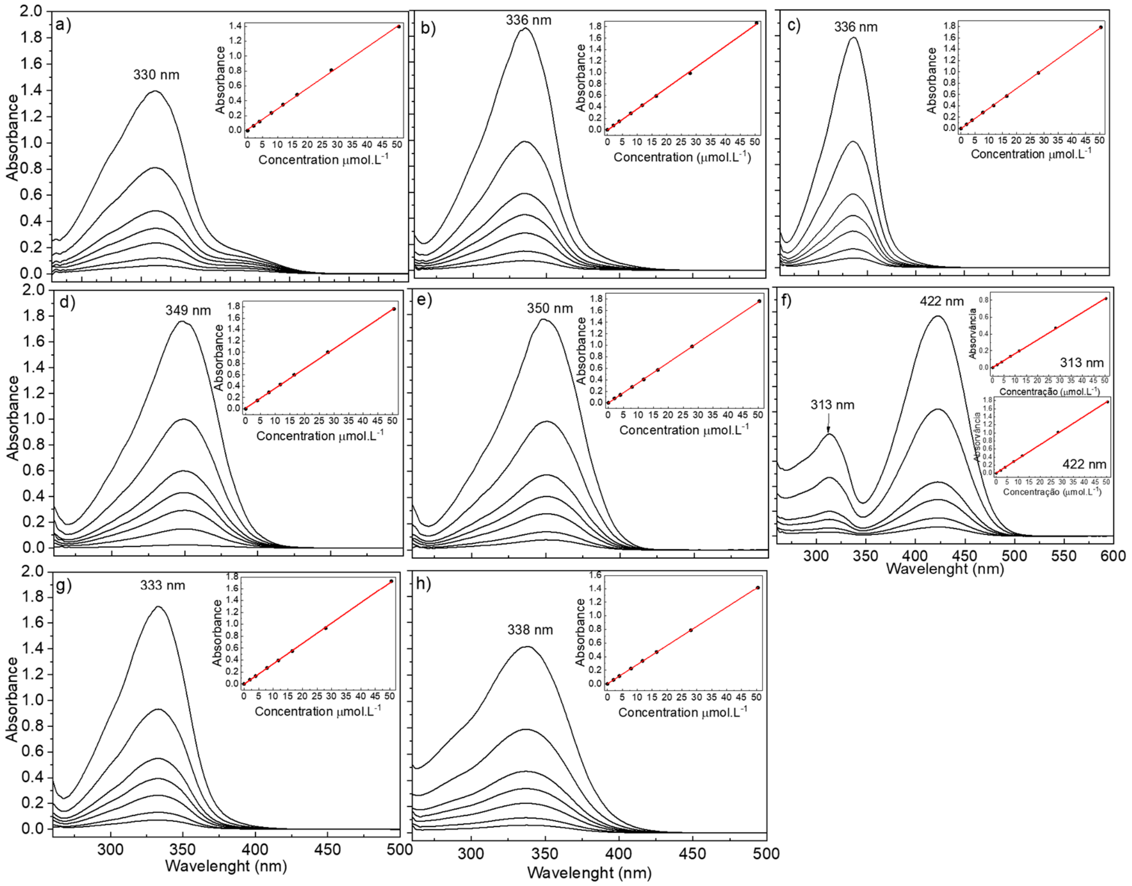

Figure S1 – Graph of absorbance as a function of molar concentration to determine the molar absorption coefficient for a)ChH b)ChCH3 c)ChCH2CH3 d)ChOCH3 e)ChOCH2CH3 f)ChN(CH3)2 g)ChBr and h)ChNO2. The insets show the linear behavior of maximum absorbance with the molar concentration.

3) Gaussian decomposition

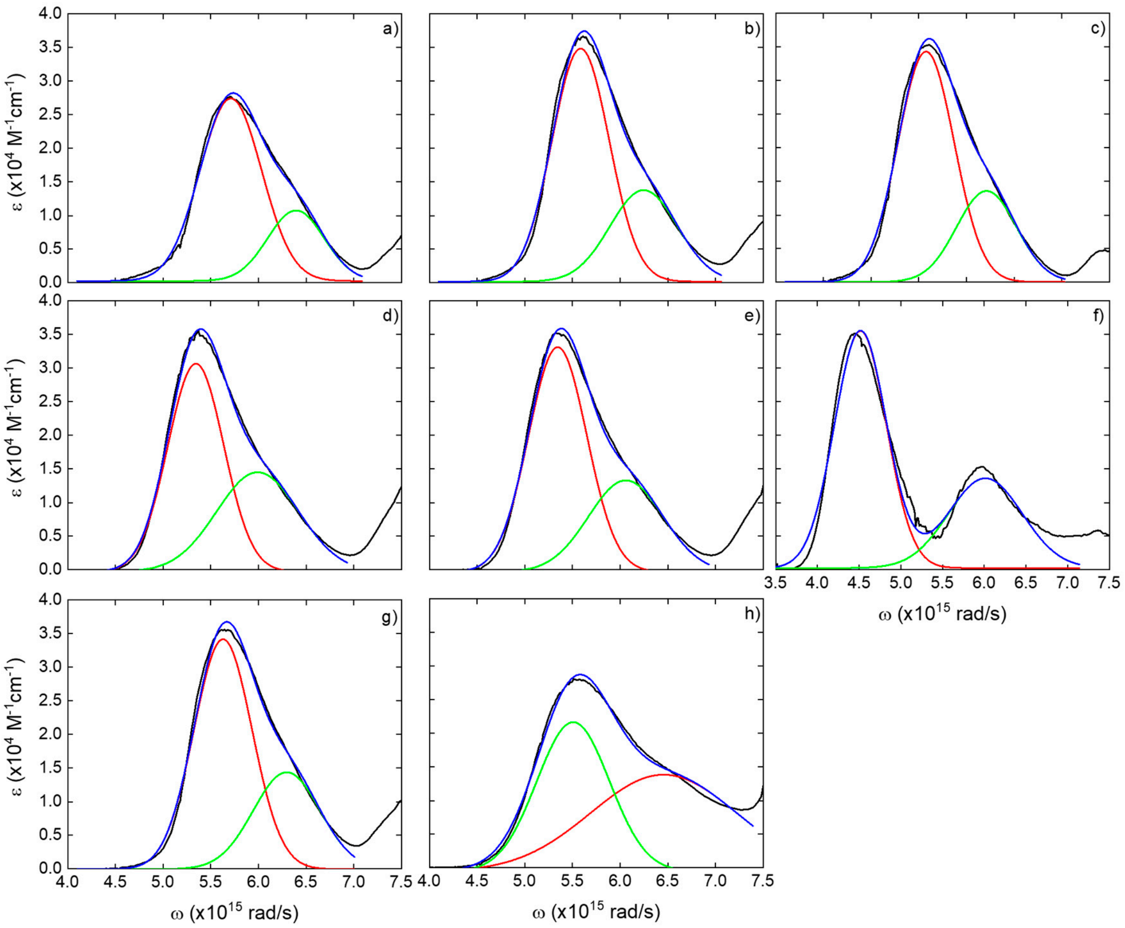

Figure S2 – Gaussian decomposition of of a)ChH b)ChCH3 c)ChCH2CH3 d)ChOCH3 e)ChOCH2CH3 f)ChN(CH3)2 g)ChBr and h)ChNO2. The red, green, and blue lines represent the first electronic state, the second electronic state, and the fitting, considering that both are overlapped.

Transition dipole moments from the ground to the first and second excited states were determined through the following equation:

$$|\vec{\mu}_{0n}|^2 = \frac{3 \times 10^3 ln\,(10)\, hc}{(2\pi)^3 N_A} \frac{n}{L^2} \frac{1}{\omega_{0n}} \int_{\varepsilon_1}^{\varepsilon_f} \varepsilon(\omega) d\omega \qquad \text{SI1}$$

in which h is the Planck constant, n is the refractive index of DMSO, L is the Onsager's field factor given by $3n^2/(2n^2+1)$, N_A is the Avogadro number, ω_{0n} is the angular transition frequency of ground (subindex 0) to excited states (n = 1,2) and $\int_{\varepsilon_1}^{\varepsilon_f} \varepsilon(\omega) d\omega$ is the area bellow each electronic band.

4) Quantum Chemical Calculations

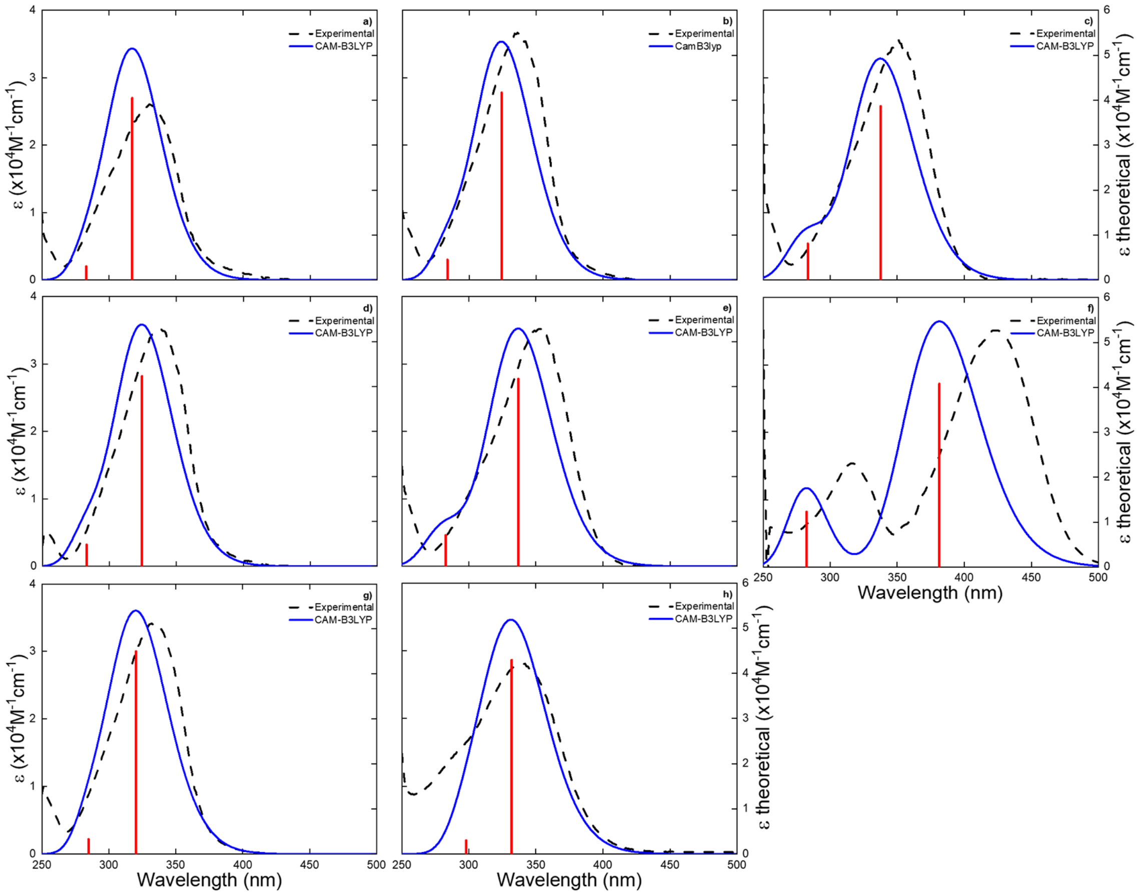

Figure S3 – Experimental (dashed black lines) and simulated (continuous blue lines) one-photon absorption spectra of a) ChH b) ChCH3 c) ChOCH3 d) ChCH2CH3 e) ChOCH2CH3 f) ChN(CH3)2 g) ChBr and h) ChNO2. The vertical red bars represent the oscillator strengths of the electronic transitions as determined via PCM-TD-DFT calculations.

5) Quantum Chemistry Calculations (QCCs)

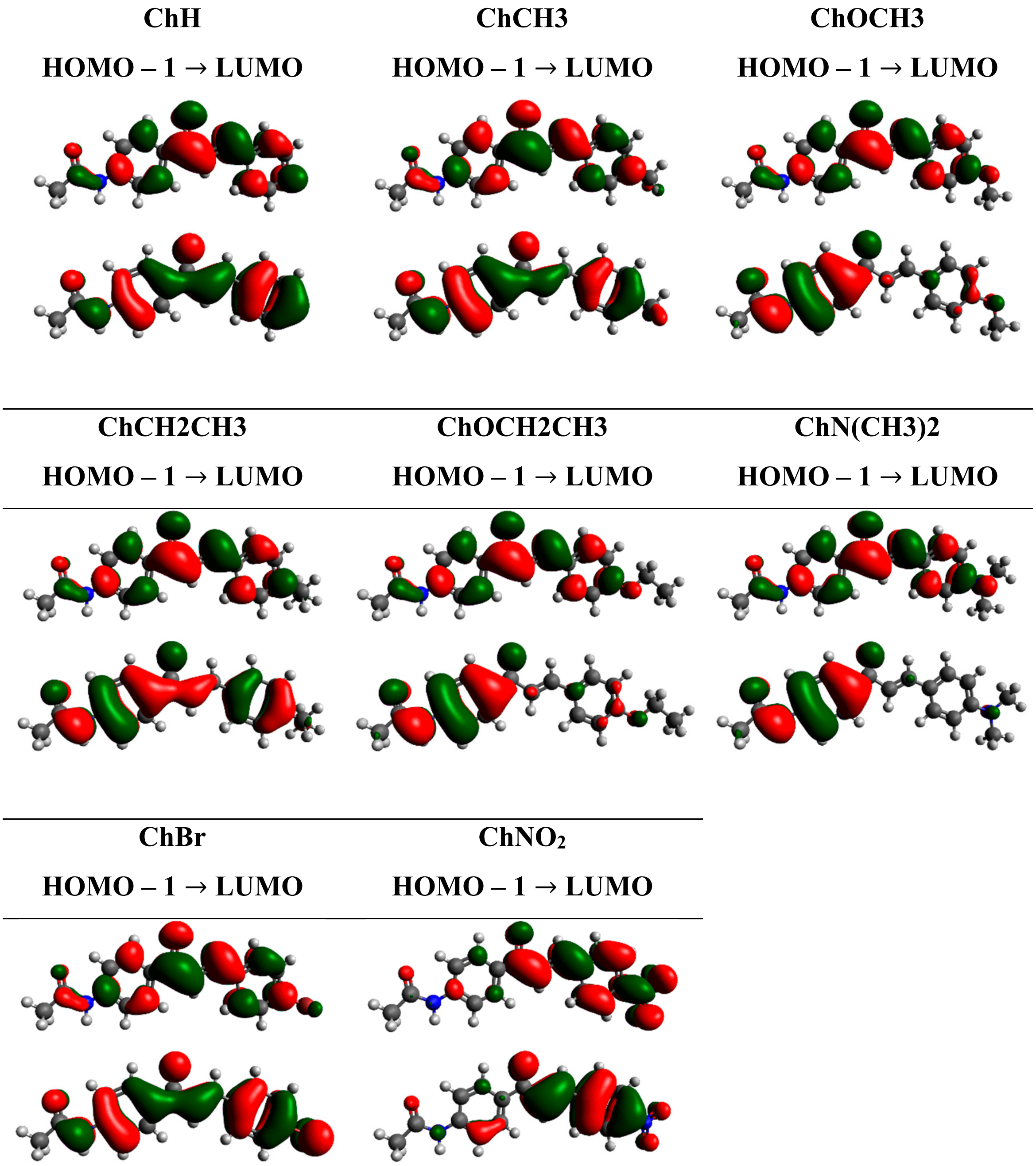

Figure S4 - Frontier Molecular obtained through the PCM-CAM-B3LY/6-311++G(d,p) approach for DMSO medium. The FMOs are involved in the lowest-energy one-photon allowed transition of all studied compounds.

Table S1 – Oscillator strengths and transition wavelengths for all Acetamide-chalcone derivatives obtained by TD-DFT calculations using the PCM-CAM-B3LYP/6-311G++(d,p) approach for DMSO medium.

Compound	$f_{S_0 \to S_1}$	$\lambda_{S_0 \to S_1}(nm)$	$f_{S_0 \to S_2}$	$\lambda_{S_0 \to S_2}(nm)$
**ChH**	1.35	317	0.10	283
**ChCH3**	1.39	324	0.14	284
**ChOCH3**	1.29	338	0.27	283
**ChCH2CH3**	1.41	324	0.16	283
**ChOCH2CH3**	1.39	337	0.23	283
**ChN(CH3)2**	1.36	381	0.41	282
**ChBr**	1.50	320	0.11	285
**ChNO2**	1.43	332	0.10	298

6) Structural analysis

Eight *para*-substituted aceto-amidechalcones were prepared by Claisen-Schmidt reaction with moderated yields (30 -74 %), and their structure was supported by spectroscopic techniques. FTIR spectra of 4-acetamideacetophenone present absorption bands in 3296 cm^{-1} due to stretching (υ) of N-H bond and a large band at 1674 cm^{-1}, related to C=O stretchings of amide and ketone groups. In sequence, $ChCH_3$ shows similar υ(N-H) absorption band and, additionally, the υ(C=O) of carbonylic groups has been split into two new bands, at 1678 cm^{-1} (amide group) cm^{-1} and 1650 cm^{-1} (ketone α,β-insaturated). Furthermore, ChCH3 exhibits a new band at 808 cm^{-1} relative to C-H bending of *para*-substituted aromatic ring resulting from the connection with 4-methylbenzaldehyde.

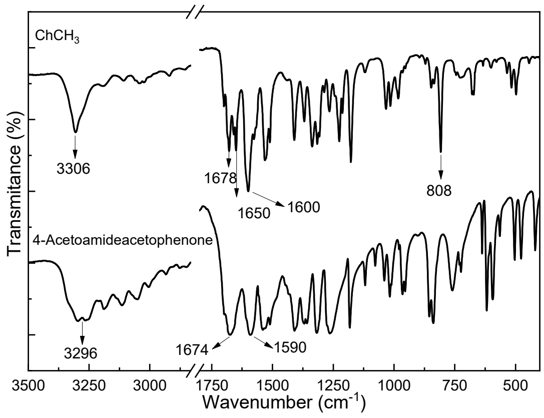

Figure S5 - FTIR Spectra of 4-Acetamideacetophenone and ChCH3

^1H NMR of all synthesized molecules shows two typical dublets of *trans*-chalcones with coupling constants of 15 Hz. These signals, near 7.4 and 7.7 ppm, are related to the magnetic coupling of α,β-olefinic hydrogens at *E* configuration. Considering the chalcone **ChCH3** (Fig SI6) are also observed two doublets, with an integral relative to 2 H, at 7.20 and 7.51 ppm (J = 8.0 Hz) associated with the hydrogens bonded to the aromatic carbons C3/C5 and C2/C6, respectively, and at 7.66 and 7.99 ppm associated with hydrogens attached to C3'/C5' and C2'/C6' aromatic carbons. On the other hand, at 2.20 and 2.37 ppm two singlets, with an integral relative to 3 hydrogens, were observed and corresponding to methylenic hydrogens of the methyl group of C8 and C9. The hydrogen of amine group consists of a large singlet at 7.84 ppm.

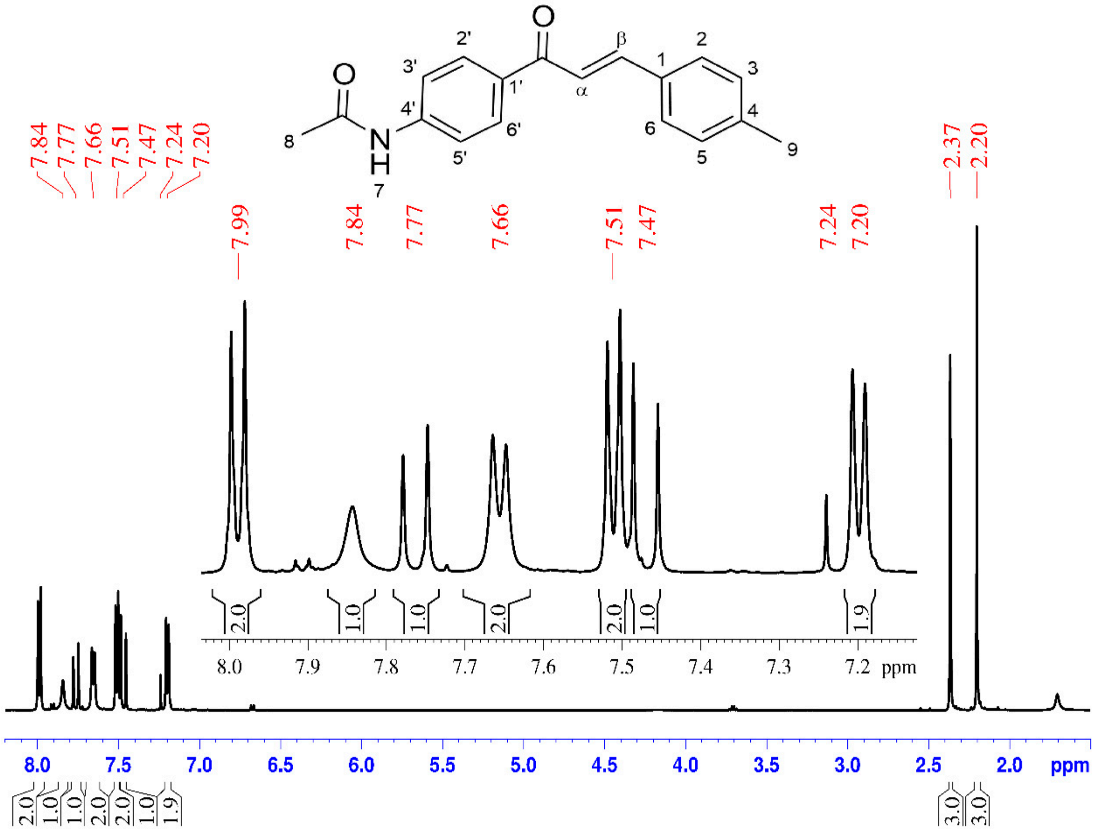

Figure S6 - ^{1}H NMR spectra, in $CDCl_3$ (7.24 ppm), of **ChCH3**.

^{13}C NMR spectra of **ChCH3** present signals in 120.8 ppm and 144.7 ppm related to carbons on α and β positions, respectively, in 168.6 ppm associated to carbonyl of amide group and 189.2 to carbonyl group of α,β-unsaturated ketone.

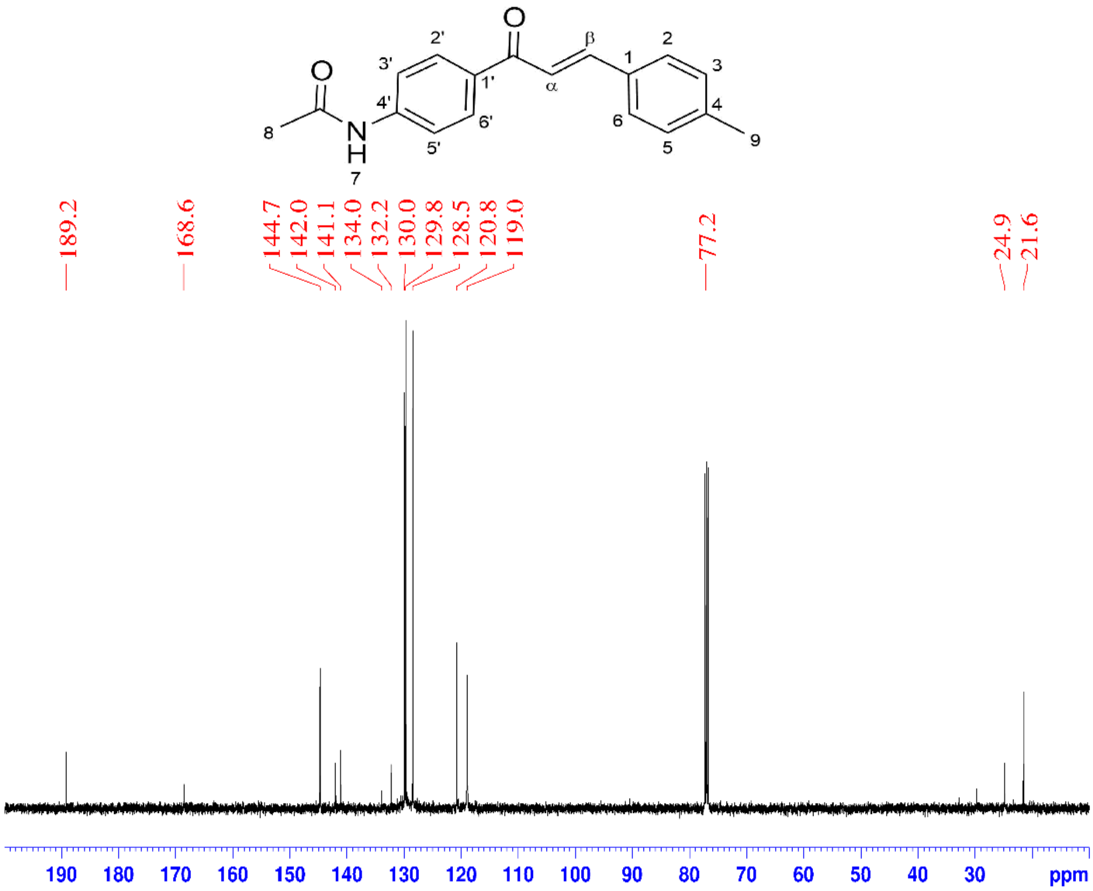

Figure S7 – ^{13}C NMR spectra, in $CDCl_3$ (77.2 ppm), of **ChCH3**.

7) FTIR spectra of all acetamide-chalcones synthesized.

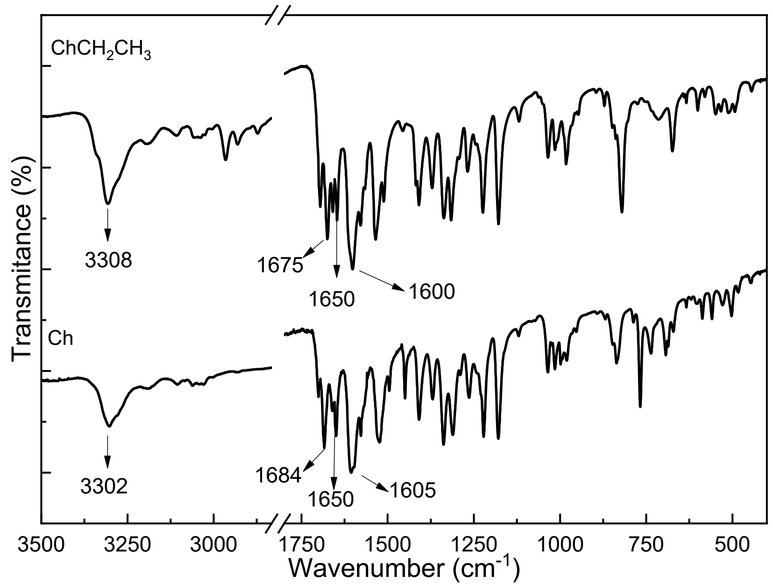

Figure S8 - FTIR Spectra of **ChH** and **ChCH2CH3**

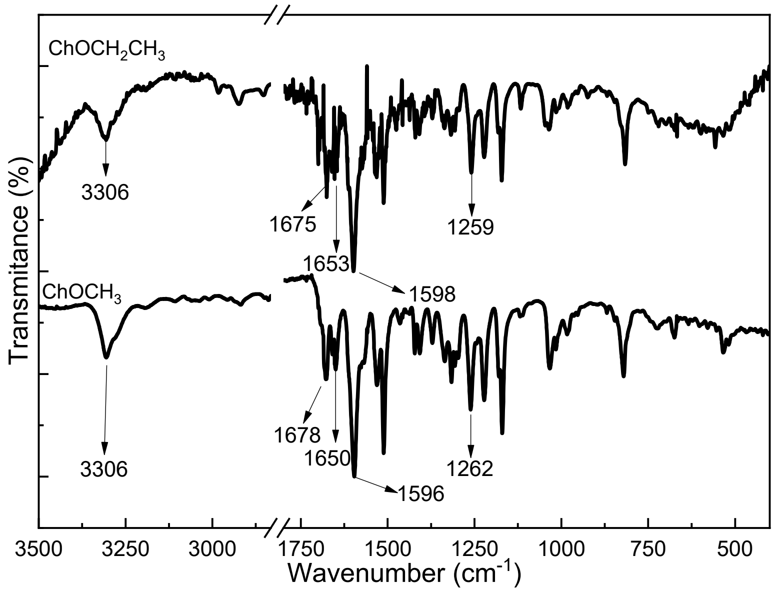

Figure S9 - FTIR Spectra of **ChOCH3** and **ChOCH2CH3**

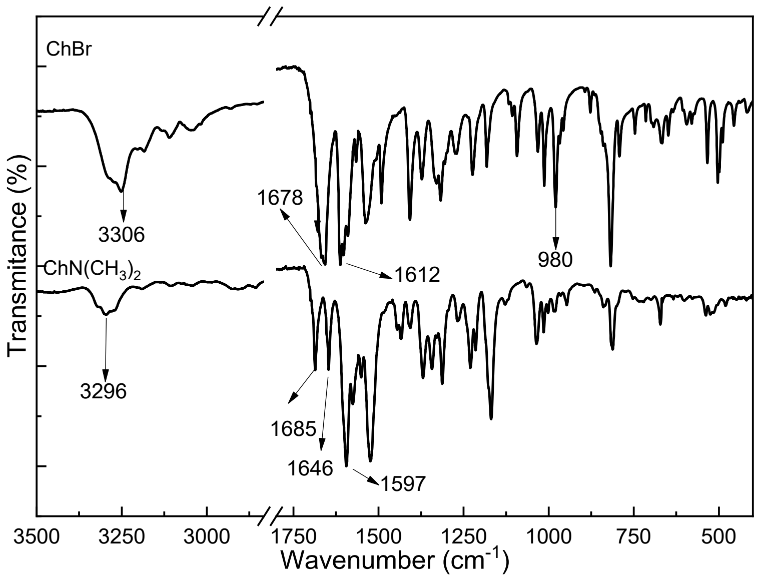

Figure S10 - FTIR Spectra of **ChN(CH3)2** and ChBr

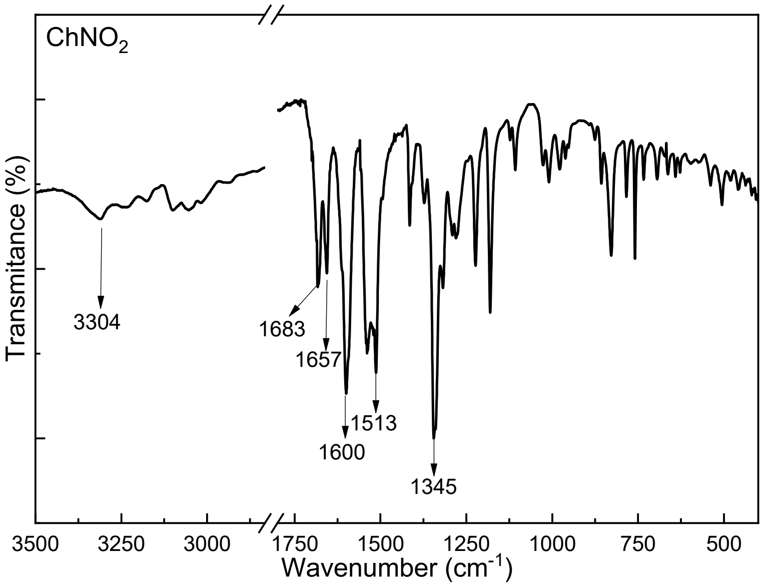

Figure S11 - FTIR Spectra of **ChNO2**

8) ¹H NMR Spectra of all studied compounds

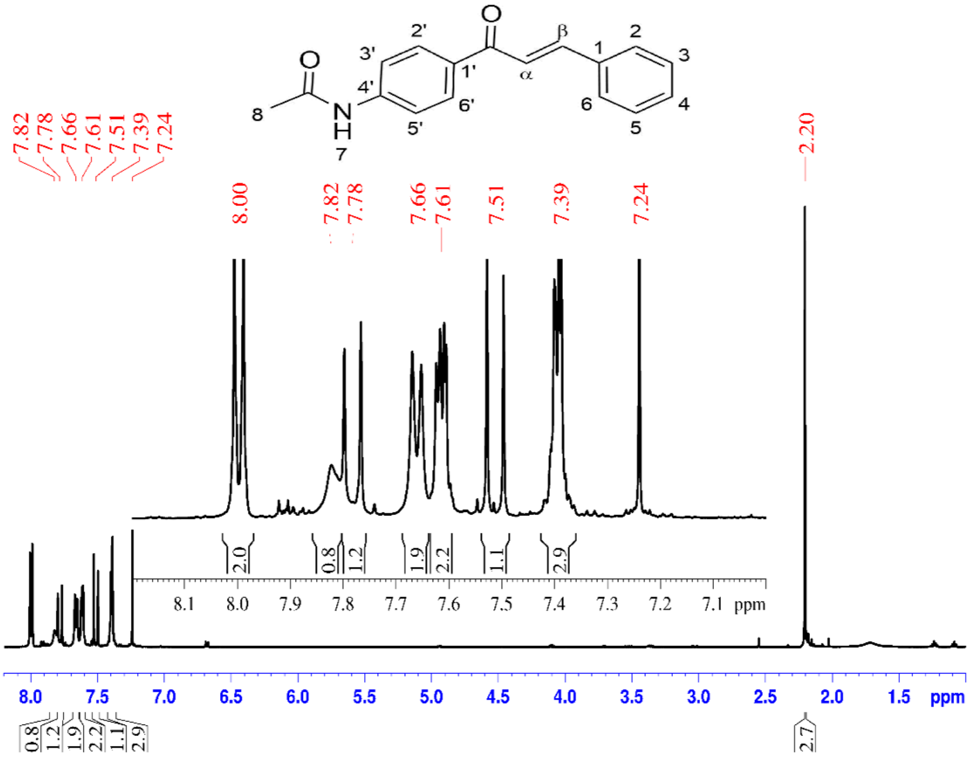

Figure S12 - ¹H NMR spectra, in $CDCl_3$ (7.24 ppm), of **ChH**.

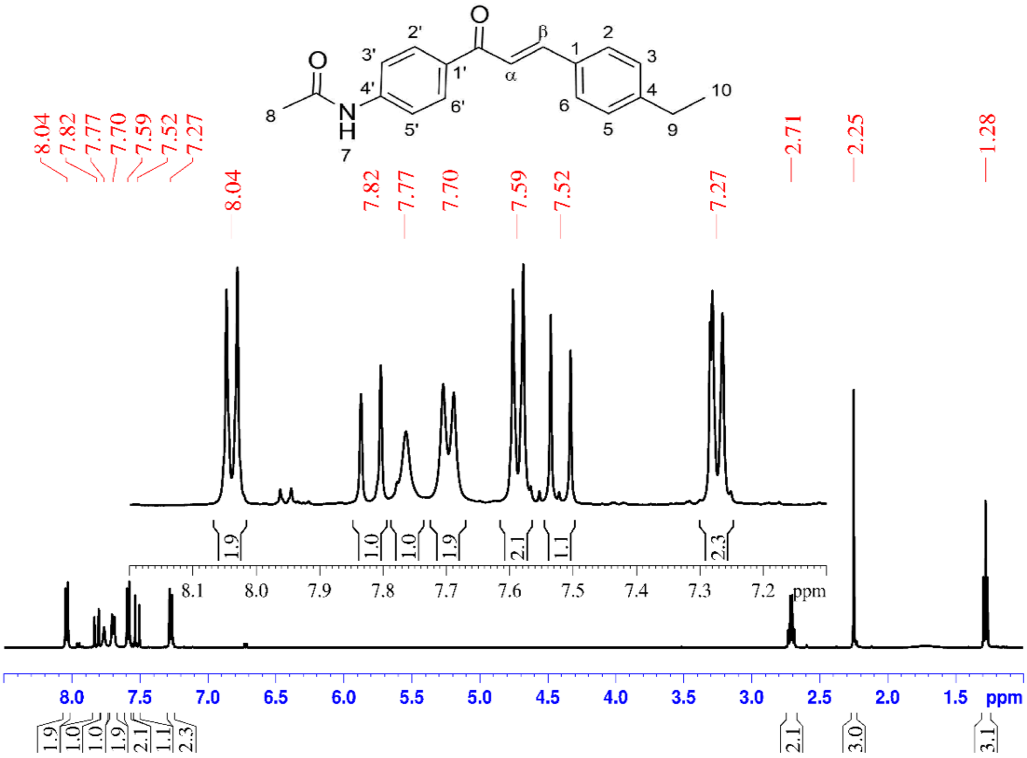

Figure S13 - ¹H NMR spectra, in $CDCl_3$ (7.24 ppm), of **ChCH2CH3**.

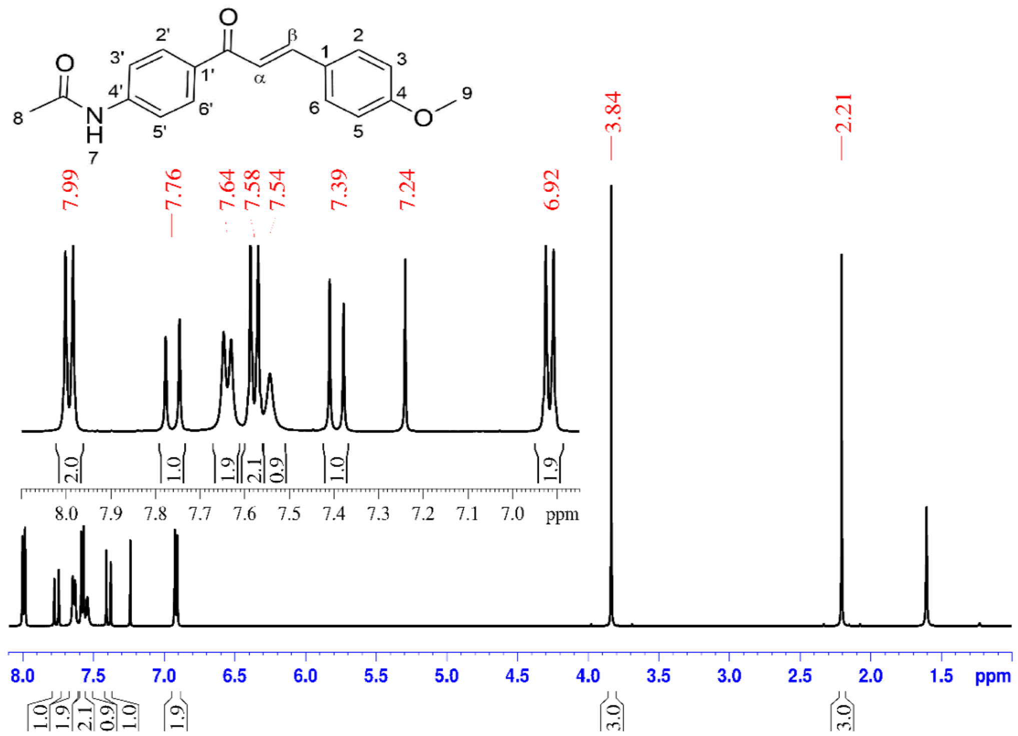

Figure S14 - ^{1}H NMR spectra, in CDCl_3 (7.24 ppm), of **ChOCH3**

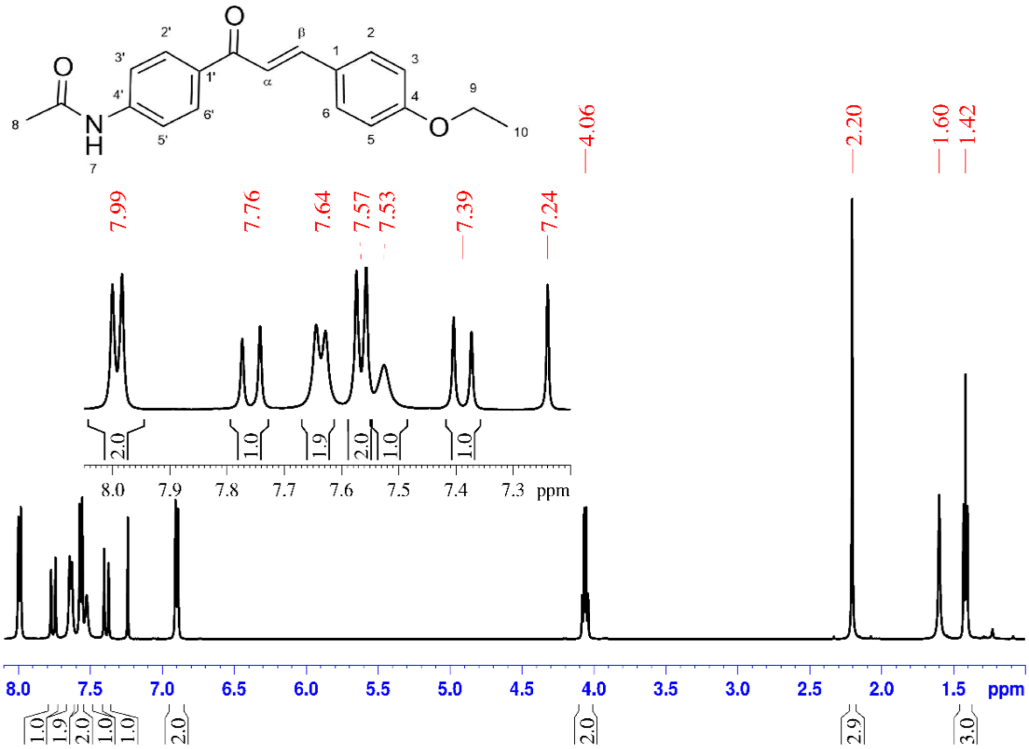

Figure S15 - ^{1}H NMR spectra, in CDCl_3 (7.24 ppm), of **ChOCH2CH3**

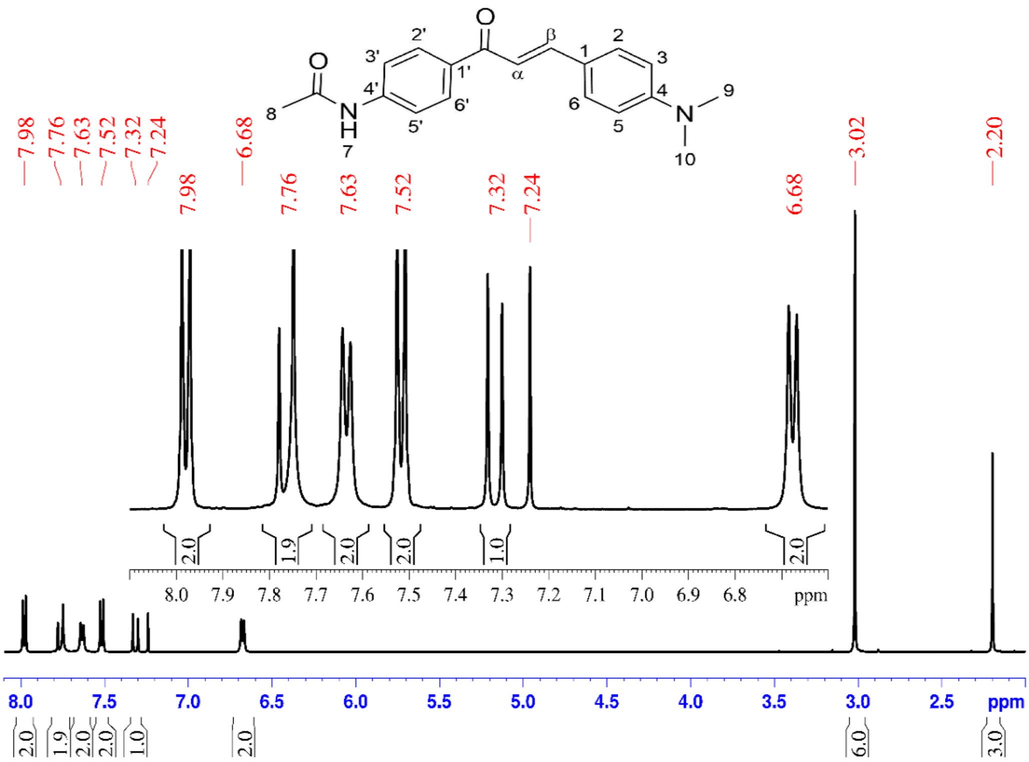

Figure S16 - ^{1}H NMR spectra, in CDC*l*$_3$ (7.24 ppm), of **ChN(CH3)2**

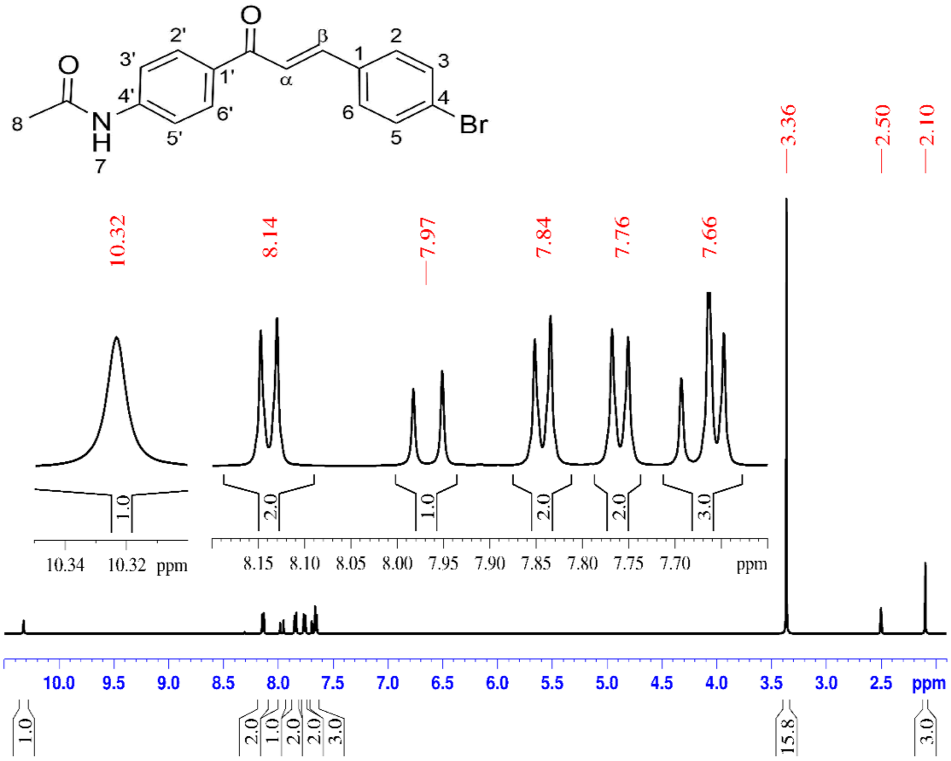

Figure S17 - ^{1}H NMR spectra, in DMSO-*d*$_6$ (2.50 ppm), of **ChBr**.

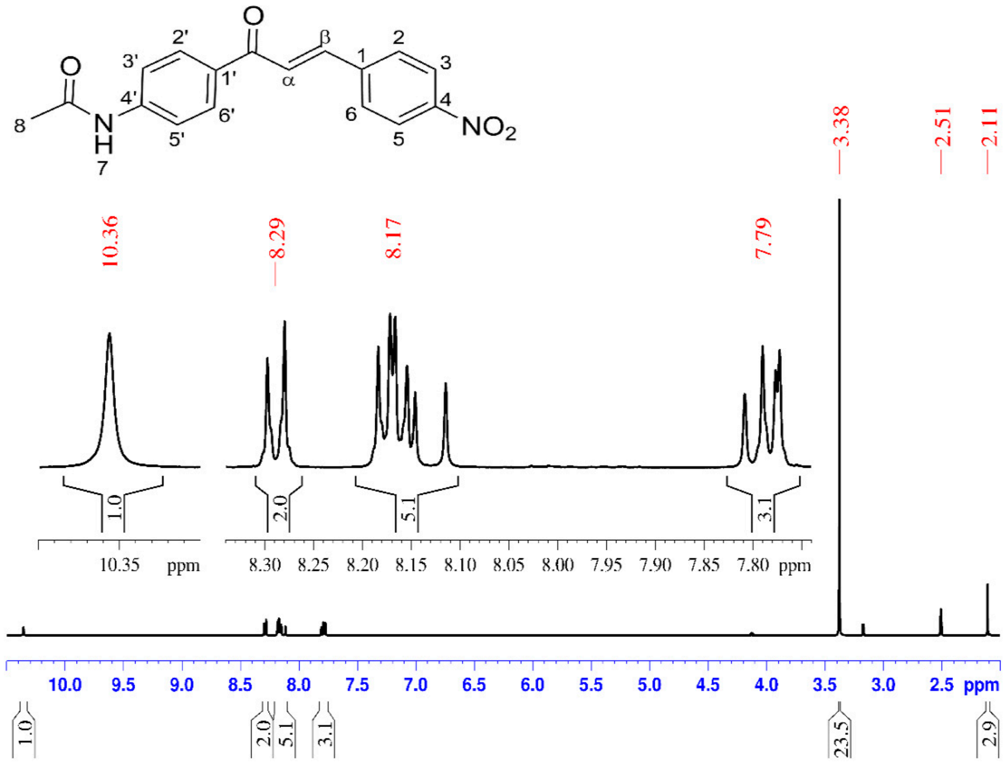

Figure S18 - ^{1}H NMR spectra, in DMSO-d_6 (2.50 ppm), of **ChNO2**.

9) ^{13}C NMR Spectra of all studied compounds.

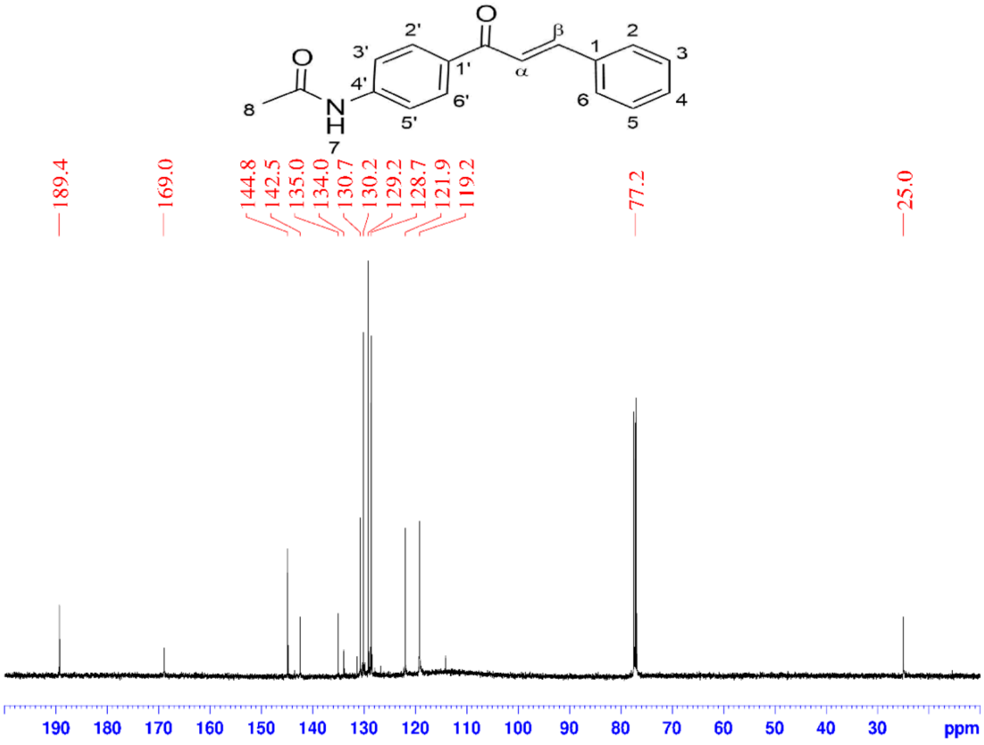

Figure S19 – ^{13}C NMR spectra, in $CDCl_3$ (77.2 ppm), of **ChCH**

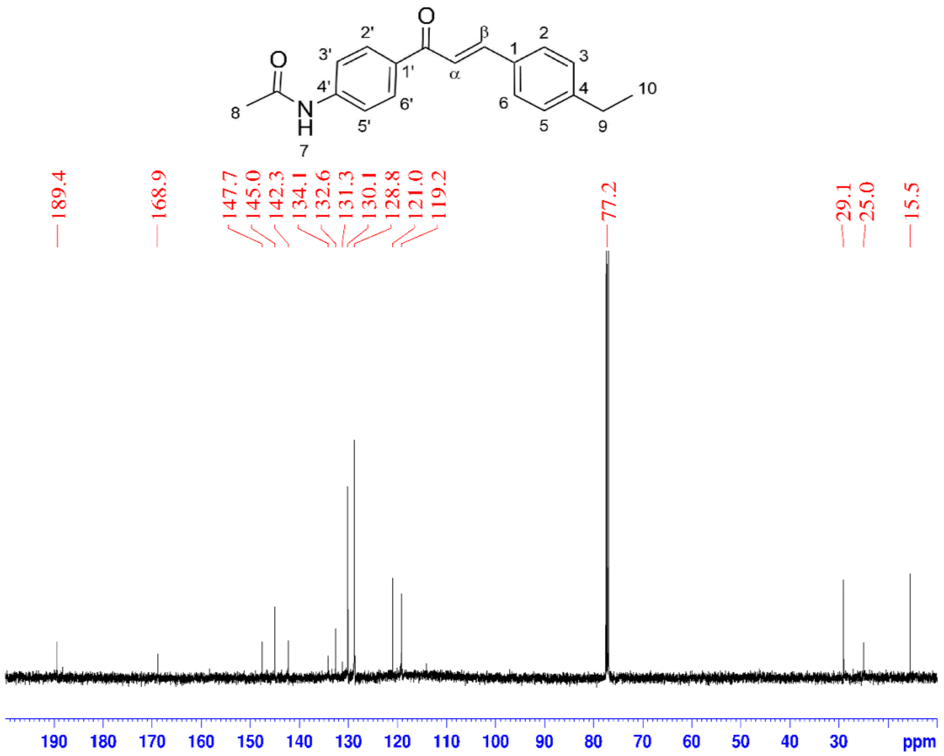

Figure S20 – ^{13}C NMR spectra, in $CDCl_3$ (77.2 ppm), of **ChCH2CH3**

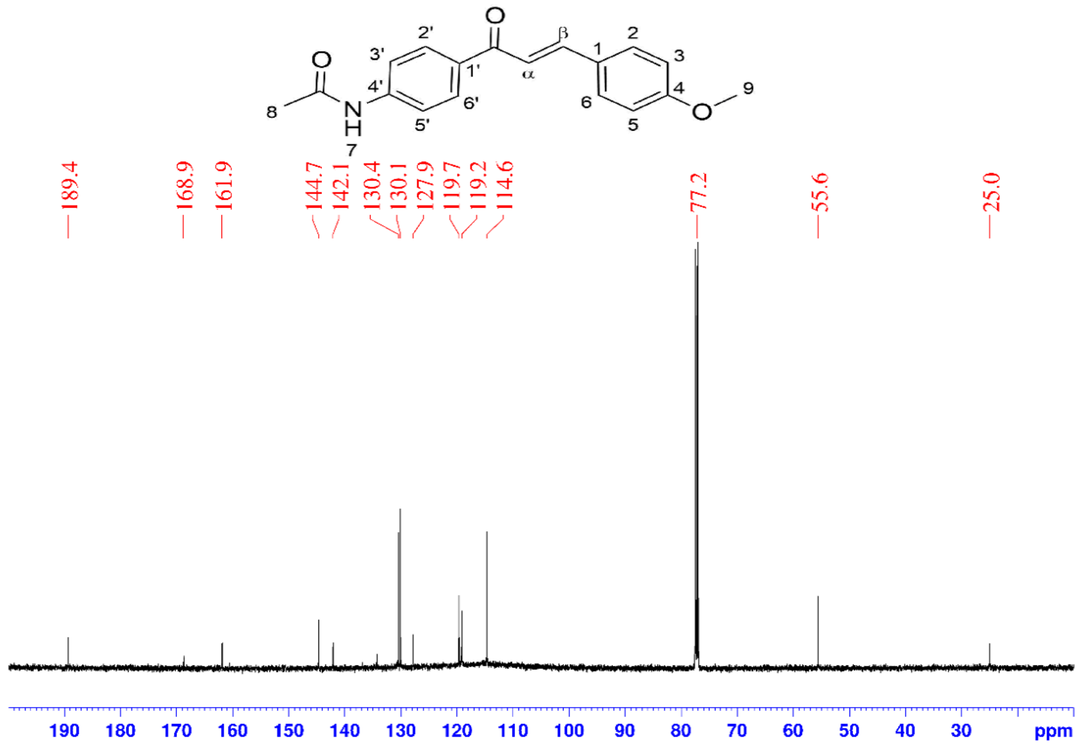

Figure S21 – ^{13}C NMR spectra, in CDCl3 (77.2 ppm), of **ChOCH3**

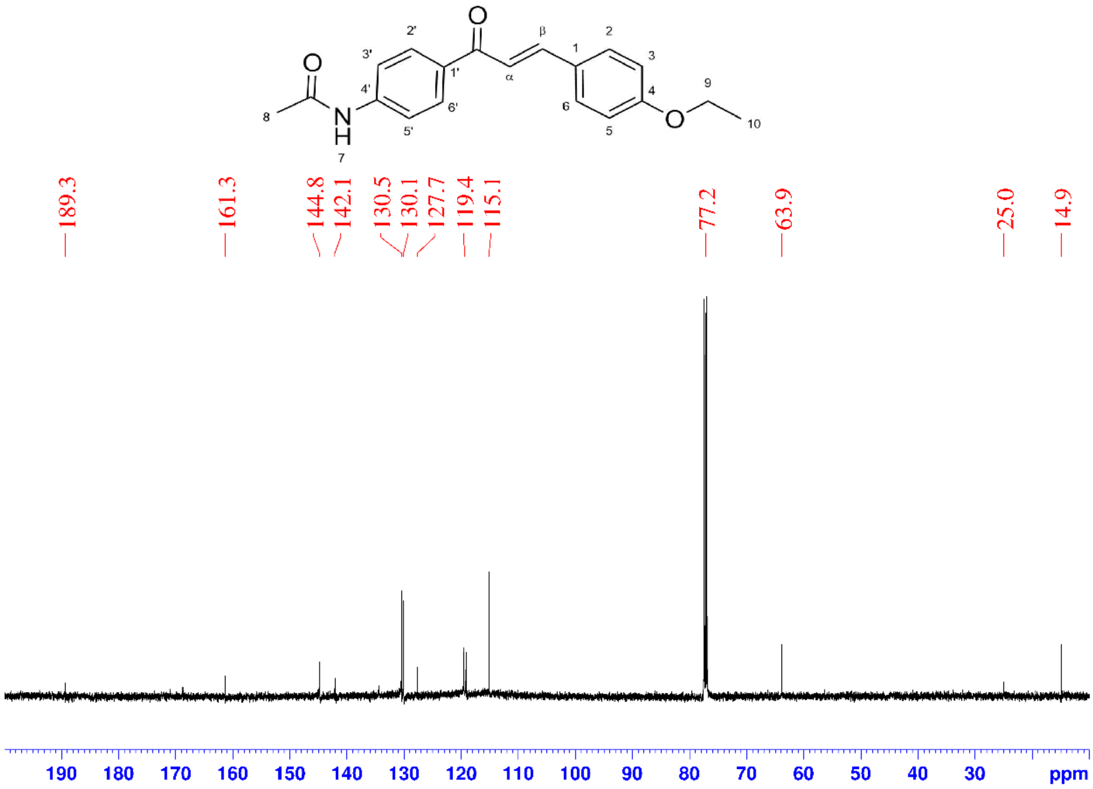

Figure S22 – ^{13}C NMR spectra, in CDCl3 (77.2 ppm), of **ChOCH2CH3**

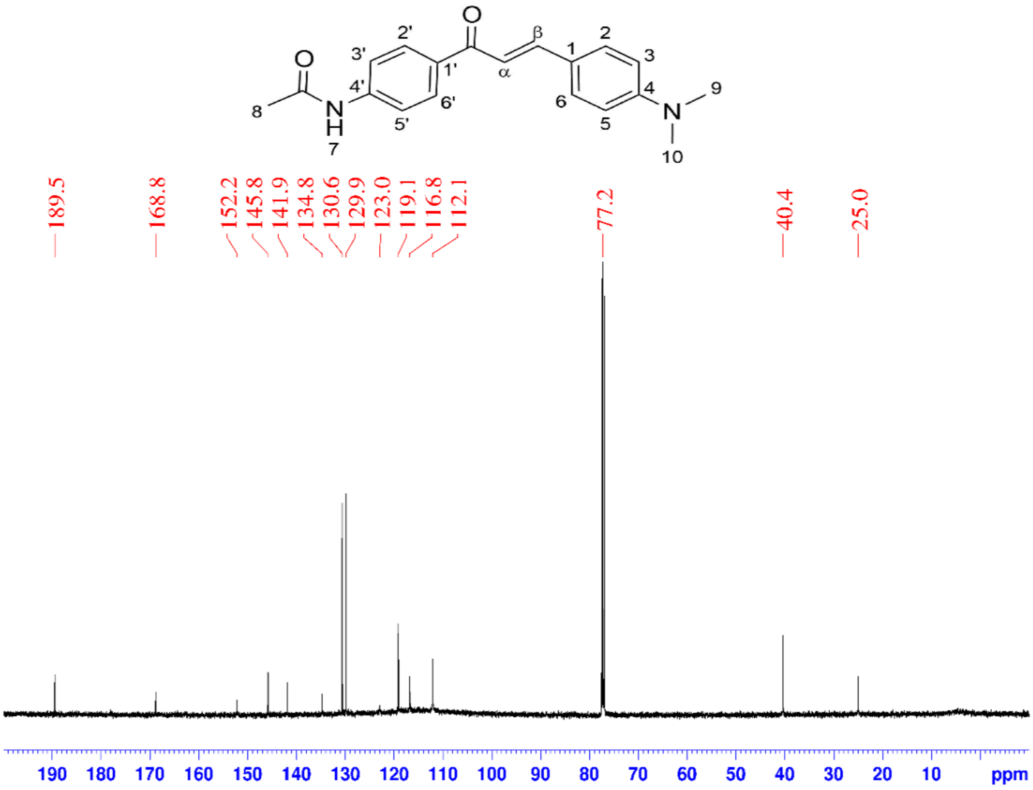

Figure S23 – ^{13}C NMR spectra, in $CDCl_3$ (77.2 ppm), of **ChN(CH3)2**

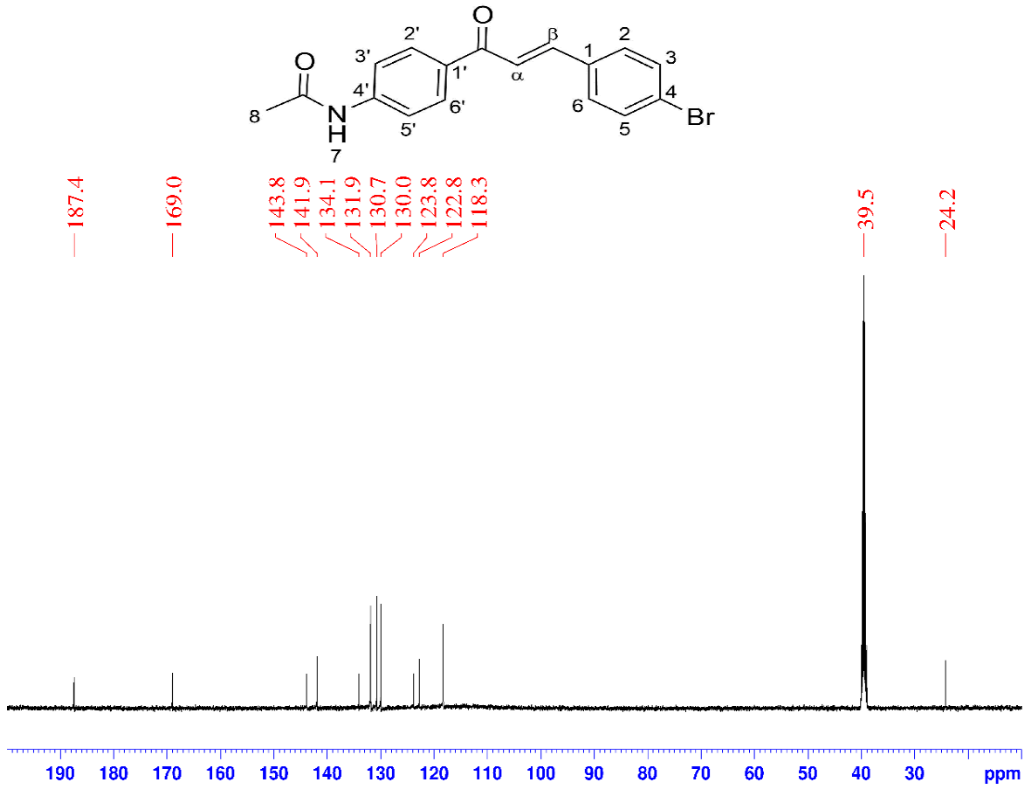

Figure S24 – ^{13}C NMR spectra, in DMSO-*d6* (39.5 ppm), of **ChBr**

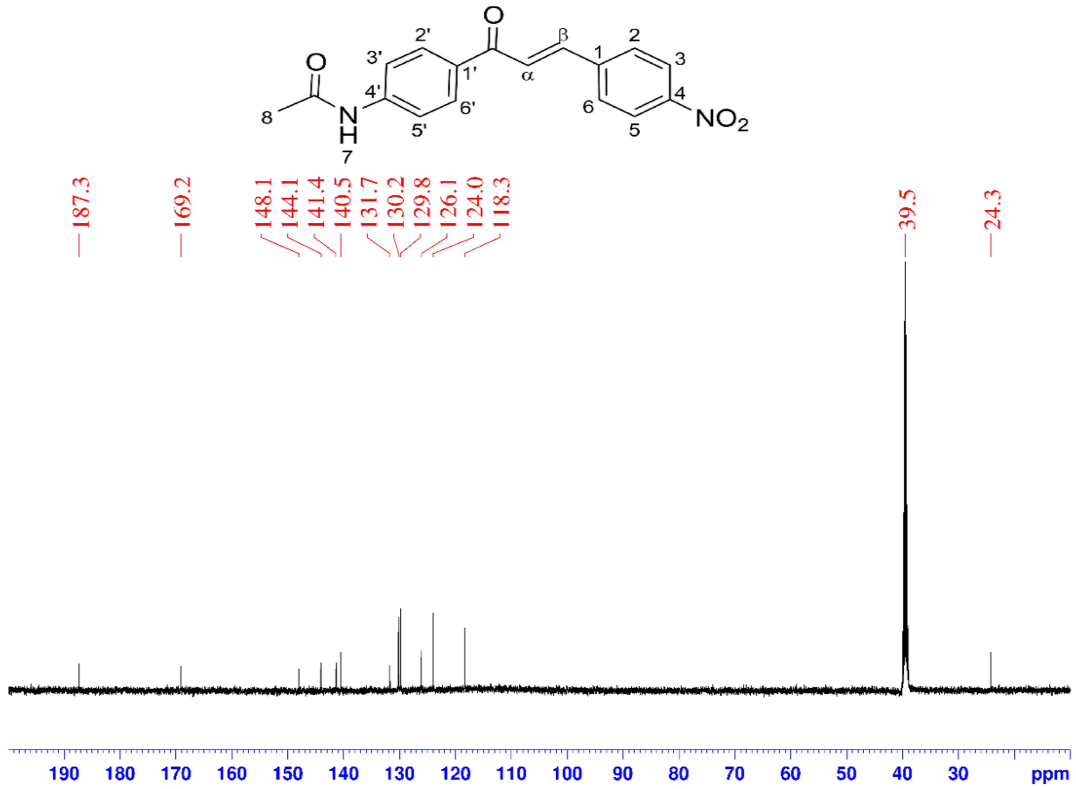

Figure S25 – ^{13}C NMR spectra, in DMSO-*d6* (39.5 ppm), of **ChNO2**

10) High-Resolution Mass Spectra Data of all studied compounds

Mass spectra were obtained using a Q Exactive hybrid Quadrupole-Orbitrap mass spectrometer (Thermo Scientific, San Jose, U.S.A). All HRESI-MS analyses were performed in positive ionization mode with the capillary temperature maintained at 320 °C, spray voltage at 4.5 kV, S-lens RF level at 60%, resolution at 140 000, three micro scans, and collision energy for MS/MS (higher-energy collisional dissociation − HCD) ranging from 20 to 30 (manufacturer's unit). The spectra were processed using the Xcalibur Analysis software package (version 2.0, Service Release 2, Thermo Electron Corporation. The HRESI-MS spectra are presented in Fig. S26-S32. All synthesized compounds showed low $[M+H]^+$ ion mass error, confirming the veracity of the structural proposition of the products.

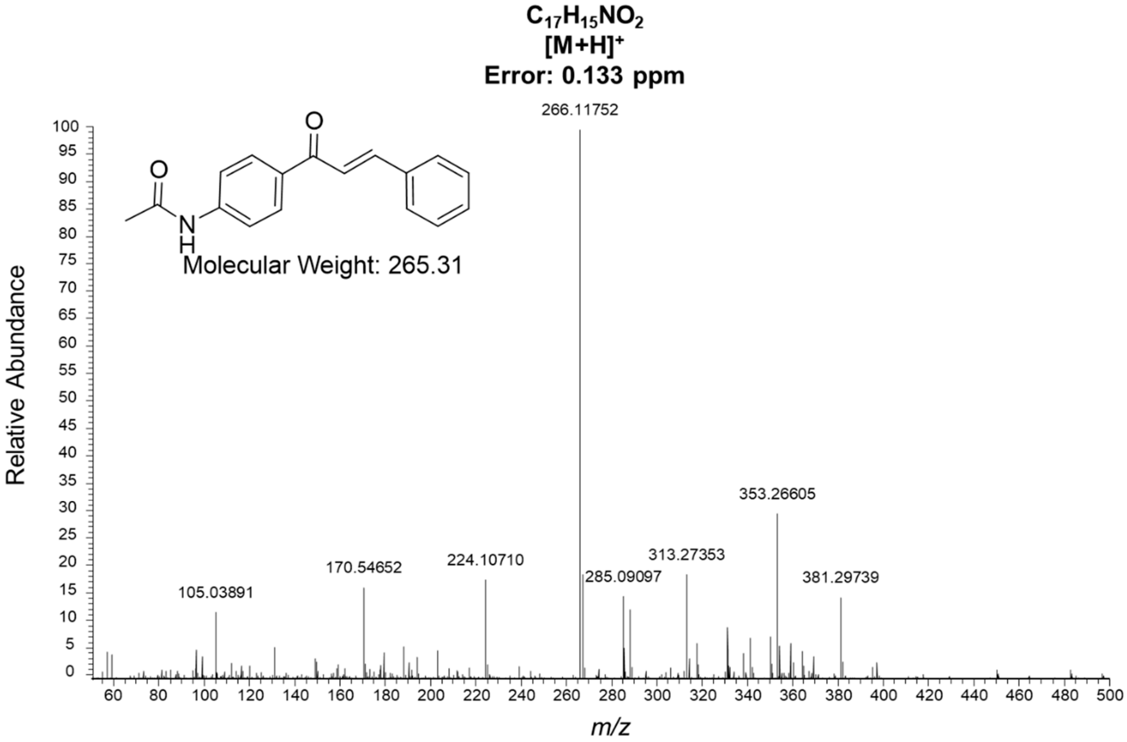

Figure S26 – HRESI-MS spectra of **ChH.**

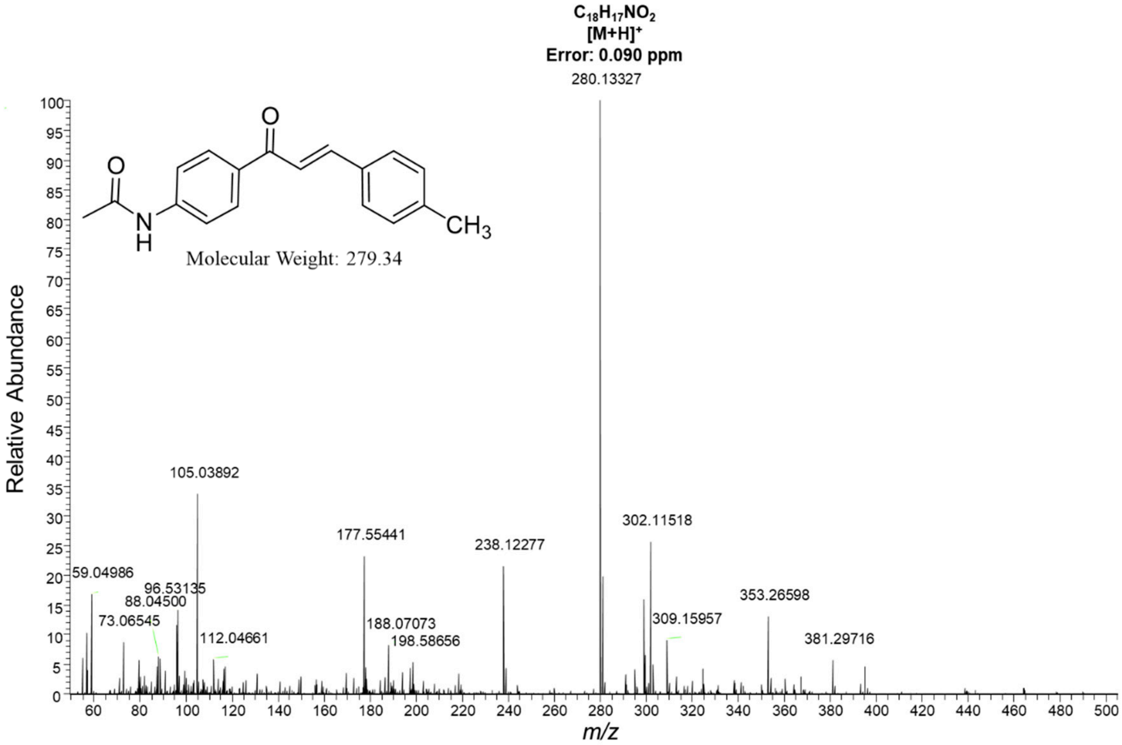

Figure S27 – HRESI-MS spectra of **ChCH3.**

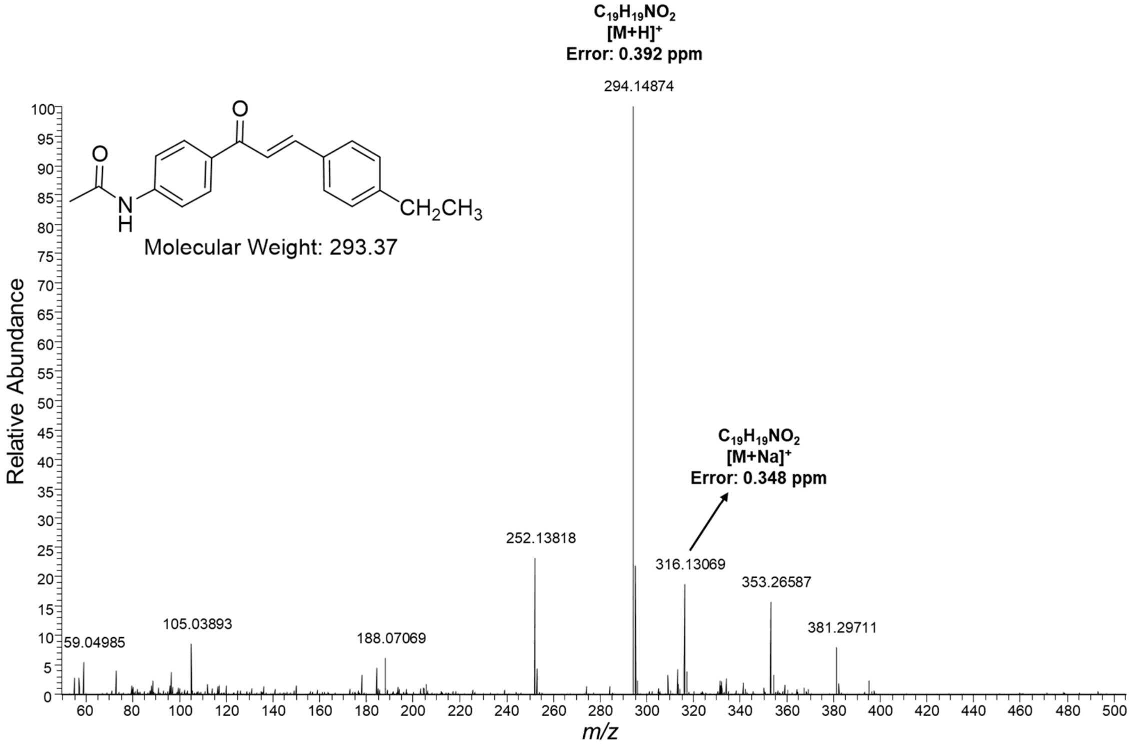

Figure S28 – HRESI-MS spectra of **ChCH2CH3.**

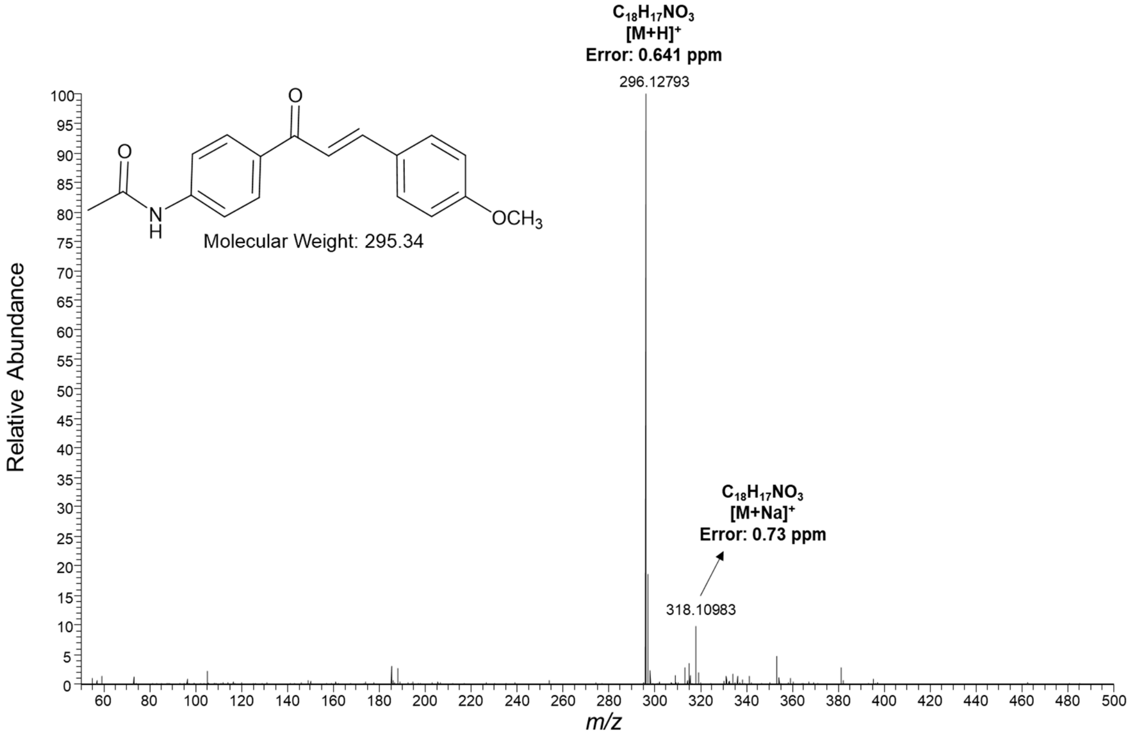

Figure S29 – HRESI-MS spectra of **ChOCH3.**

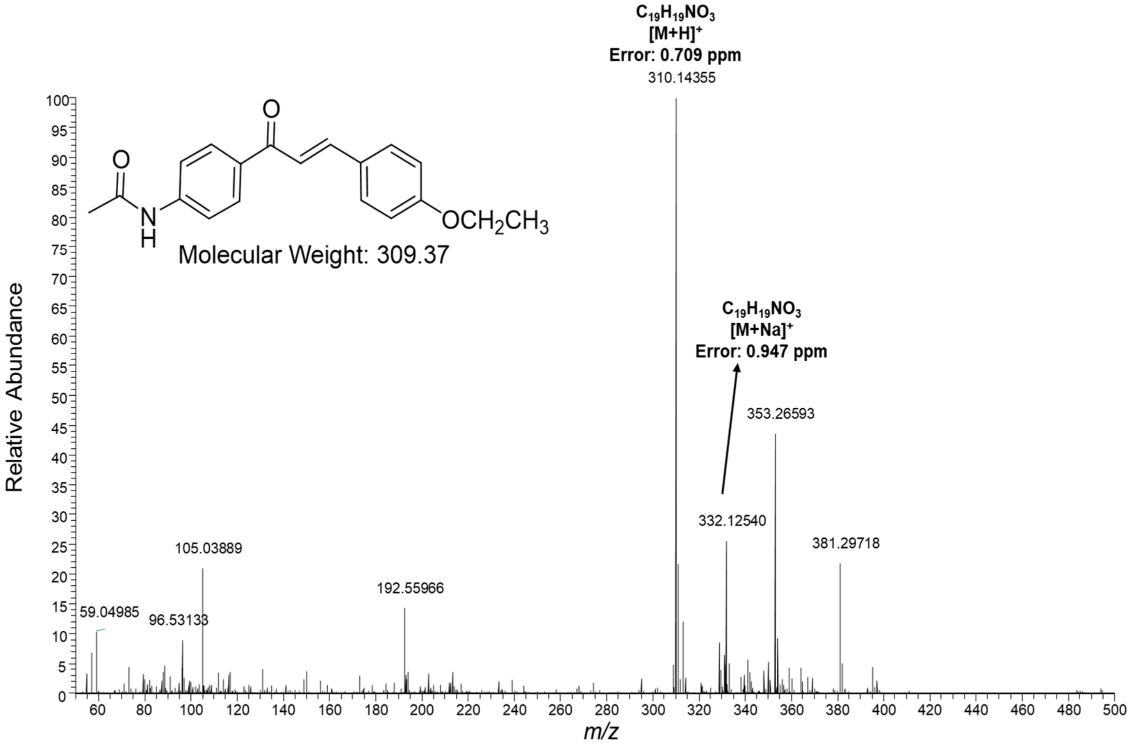

Figure S30 – HRMS spectra of **ChOCH2CH3.**

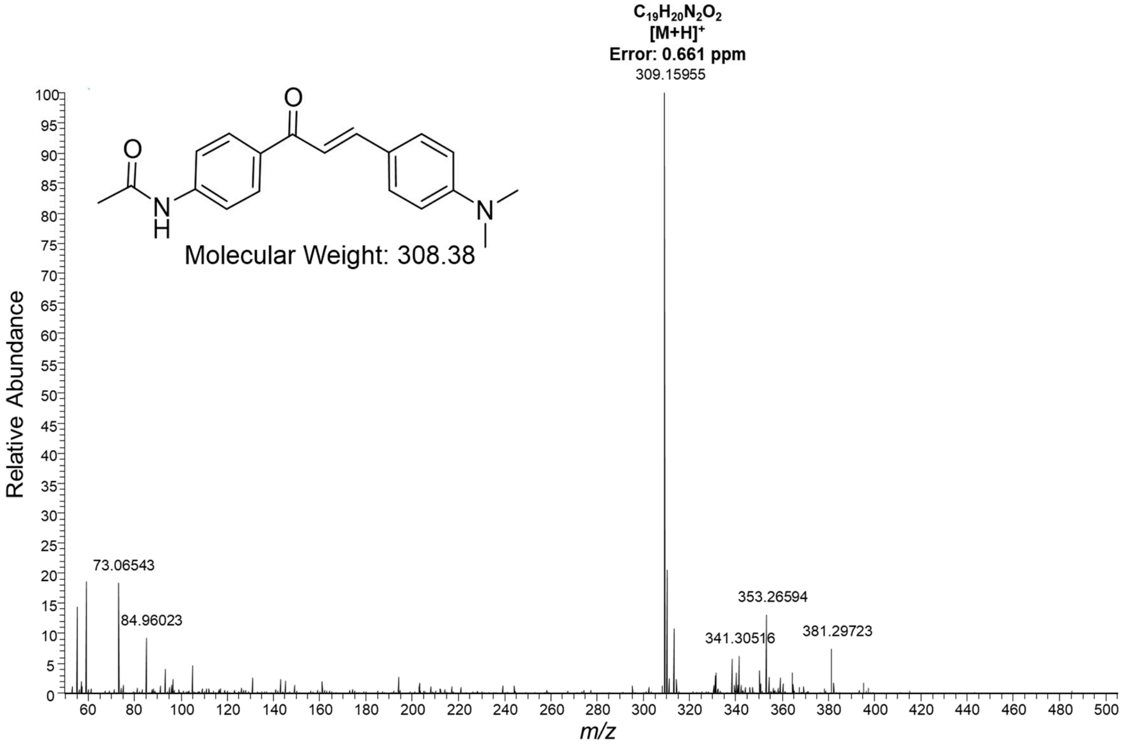

Figure S*31* – HRESI-MS spectra of **ChN(CH3)2.**

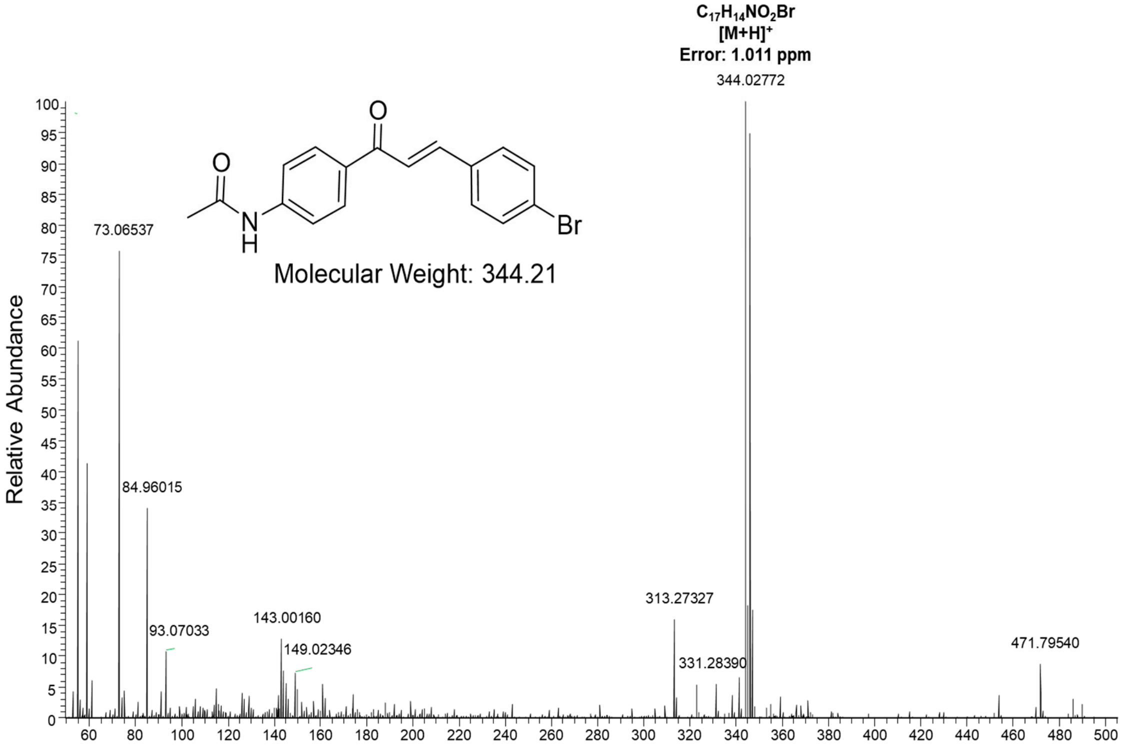

Figure S*32* – HRESI-MS spectra of **ChBr.**

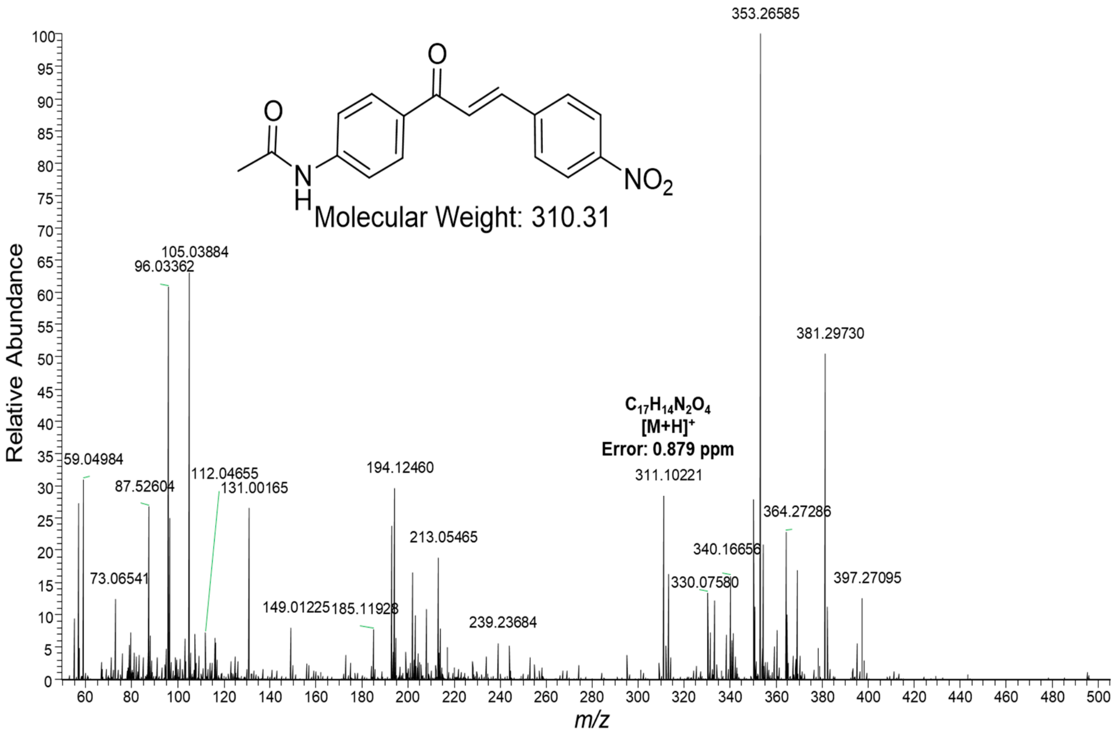

Figure S33 – HRESI-MS spectra of **ChNO2.**

11) Aditional Structural Information of all studied compounds

a) N-[4-[(2E)-1-Oxo-3-phenyl-2-propen-1-yl]phenyl]acetamide (**ChH**): $C_{17}H_{15}NO_2$; Reaction Yield: 30 %; Molecular Weight: 265,31 g.mol^{-1}; CAS Number: 123456-58-6; ε_{DMSO} (330 nm): 27716 mol^{-1}.L.cm^{-1}; HRESI-MS: $[M+H]^+$ = m/z 266.11752

FTIR (cm^{-1}): 3302 (υ -N-H); 1684 (υ -NHC=O); 1650 (υ $C_{(\alpha,\beta)}$ = O); 1605 (υ -HC_α=C_βH).

^{1}H NMR 500MHz $CDCl_3$ (δ ppm, m, J): 8.00 ppm (d, 8.6 Hz, H-2'/H-6'); 7.82 ppm (br. s, H-7); 7.78 ppm (d, 15.5 Hz, H-β); 7.66 ppm (d, 8.6 Hz, H-3'/H-5'); 7.61 ppm (m, H-2/H-6); 7.51 ppm (d, 15.5 Hz, H-α); 7.39 ppm (m, H-3/H-4/H-5); 2.7 ppm (s, 8.6, H-8).

^{13}C NMR 125MHz $CDCl_3$ (δ ppm): 25.0 ppm (C8)); 121.9 ppm (C_α); 144.8 ppm(C_β); 199.2-142.4 ppm ($C_{aromatics}$); 169.0 ppm (C=O, amide); 189.4 ppm (C=O, α-β).

b) N-[4-[(2E)-3-(4-Methylphenyl)-1-oxo-2-propen-1-yl]phenyl]acetamide (**ChCH3**): $C_{118}H_{17}NO_2$; Reaction Yield: 72 %; Molecular Weight: 279,34 g.mol^{-1}; CAS Number: 1015084-94-2; ε_{DMSO} (336 nm): 36674 mol^{-1}.L.cm^{-1}; HRESI-MS: $[M+H]^+$ = m/z 280.13327

FTIR (cm^{-1}): 3296 (υ -N-H); 1678 (υ -NHC=O); 1650 (υ $C_{(\alpha,\beta)}$ = O); 1600 (υ -HC_{α}=C_{β}H).

^{1}H NMR 500MHz $CDCl_3$ (δ ppm, m, J): 7.99 ppm (d, 8.6 Hz, H-2'/H-6'); 7.84 ppm (br. s, H-7); 7.77 ppm (d, 15 Hz, H-β); 7.66 ppm (d, 8.6 Hz, H-3'/H-5'); 7.51 ppm (d, 8.6 Hz, H-2/H-6); 7.47 ppm (d, 15.5 Hz, H-α); 7.20 ppm (d, 8.6 Hz, H-3/H-5); 2.71 ppm (q, 7.4 Hz, H-9); 2.25 ppm (s, H-8); 1,28 ppm (t, 7.6 Hz, H-10)

^{13}C NMR 125MHz $CDCl_3$ (δ ppm): 21.5 ppm (C-9)); 24.0 ppm (C-8); 120.8 ppm (C_{α}); 144.7 ppm(C_{β}); 129.8-134.0 ppm ($C_{aromatics}$); 168.0 ppm (C=O, amide); 189.0 ppm (C=O, α-β).

c) N-[4-[(2E)-3-(4-Ethylphenyl)-1-oxo-2-propen-1-yl]phenyl]acetamide (**ChCH2CH3**): $C_{19}H_{19}NO_2$; Reaction Yield 46 %; Molecular Weight 293,37 $g.mol^{-1}$; CAS Number: 1993646-89-1; ε_{DMSO} (336 nm): 35303 $mol^{-1}.L.cm^{-1}$; HRESI-MS: $[M+H]^+$ = m/z 294.14874

FTIR (cm^{-1}): 3308 (υ -N-H); 1675 (υ -NHC=O); 1650 (υ $C_{(\alpha,\beta)}$ = O); 1600 (υ -HC_{α}=C_{β}H).

^{1}H NMR 500MHz $CDCl_3$ (δ ppm, m, J): 8.04 ppm (d, 8.8 Hz, H-2'/H-6'); 7.77 ppm (br. s, H-7); 7.82 ppm (d, 15 Hz, H-β); 7.77 ppm (d, 8.6 Hz, H-3'/H-5'); 7.59 ppm (d, 8.6 Hz, H-2/H-6); 7.52 ppm (d, 15.5 Hz, H-α); 7.20 ppm (d, H-3/H-5); 2.37 ppm (s, H-8); 2.20 ppm (s, H-8)

^{13}C NMR 125MHz $CDCl_3$ (δ ppm): 25.0 ppm (C-9); 29.1 ppm (C-8); 121.0 ppm (C_{α}); 145.0 ppm(C_{β}); 119.2-147.7 ppm ($C_{aromatics}$); 168.9 ppm (C=O, amide); 189.4 ppm (C=O, α-β).

d) N-[4-[(2E)-3-(4-Methoxyphenyl)-1-oxo-2-propen-1-yl]phenyl]acetamide (**ChOCH3**): $C_{18}H_{17}NO_3$; Reaction Yield 41 %; Molecular Weight: 295,34 $g.mol^{-1}$. CAS Number: 939047-39-9; ε_{DMSO} (348 nm): 34857 $mol^{-1}.L.cm^{-1}$; HRESI-MS: $[M+H]^+$ = m/z 296.12793.

FTIR (cm^{-1}): 3306 (υ -N-H); 1678 (υ -NHC=O); 1650 (υ $C_{(\alpha,\beta)}$ = O); 1593 (υ -HC_{α}=C_{β}H); 1262 (υ C_{Ph} - O).

^{1}H NMR 500MHz $CDCl_3$ (δ ppm, m, J): 7.99 ppm (d, 8.6 Hz, H-2'/H-6'); 7.99 ppm (d, 8.6 Hz, H-2'/H-6'); 7.76 ppm (d, 15.6 Hz, H-β); 7.64 ppm (d, 8.6 Hz, H-3'/H-5'); 7.58 ppm (d, 8.6 Hz, H-2/H-6); 7.54 ppm (br. s, H-7); 7.39 ppm (d, 15.6 Hz, H-α); 6.92 ppm (d, H-3/H-5); 2.37 ppm (s, H-8); 3.84 ppm (s, H-9); 2.06 ppm (s, H-8).

^{13}C NMR 125MHz $CDCl_3$ (δ ppm): 25.0 ppm (C-8); 55.6 ppm (C-9); 119.6 ppm (C_α); 144.7 ppm(C_β); 119.1-142.7 ppm ($C_{aromatics}$); 169.0 ppm (C=O, amide); 189.4 ppm (C=O, α-β).

e) N-[4-[(2E)-3-(4-Ethoxyphenyl)-1-oxo-2-propen-1-yl]phenyl]acetamide (**ChOCH2CH3**): $C_{19}H_{19}NO_3$; Reaction Yield: 42 %; Molecular Weight: 309,37 $g.mol^{-1}$; CAS Number**:** 2378628-58-9; ε_{DMSO}(348nm): 34960 $mol^{-1}.L.cm^{-1}$; HRESI-MS: $[M+H]^+$ = m/z 310.14355

FTIR (cm^{-1}): 3306 (υ -N-H); 1678 (υ -NHC=O); 1650 (υ $C_{(\alpha,\beta)}$ = O); 1593 (υ -HC_α=C_βH); 1259 (υ C_{Ph} - O).

^{1}H NMR 500MHz $CDCl_3$ (δ ppm, m, J): 7.99 ppm (d, 8.3 Hz, H-2'/H-6'); 7.76 ppm (d, 15.5 Hz, H-β); 7.64 ppm (d, 8.3 Hz, H-3'/H-5'); 7.57 ppm (d, 8.3 Hz, H-2/H-6); 7.53 ppm (br. s, H-7); 7.39 ppm (d, 15.5 Hz, H-α); 6.92 ppm (d, 8.3 Hz, H-3/H-5); 4.06 ppm (q, 7.0 Hz, H-9); 3.84 ppm (s, H-9); 2.20 ppm (s, H-8); 1,42 ppm (t, H-10).

^{13}C NMR 125MHz $CDCl_3$ (δ ppm): 25.4 ppm (C-8); 63.9 ppm (C-9); 119.4 ppm (C_α); 144.8 ppm(C_β); 115.1-144.8 ppm ($C_{aromatics}$); 169.0 ppm (C=O, amide); 189.4 ppm (C=O, α-β).

f) N-[4-[(2E)-3-[4-(Dimethylamino)phenyl]-1-oxo-2-propen-1-yl]phenyl]acetamide (**ChN(CH3)2**): $C_{19}H_{20}N_2O_2$; Reaction Yield: 30 %; Molecular Weight: 308,38 $g.mol^{-1}$; CAS Number: 1015084-98-6; ε_{DMSO} (31 3nm): 16331 $mol^{-1}.L.cm^{-1}$; ε_{DMSO}(422nm): 35169 $mol^{-1}.L.cm^{-1}$; HRESI-MS: $[M+H]^+$ = m/z 309.15955

FTIR (cm^{-1}): 3296 (υ -N-H); 1685 (υ -NHC=O); 1646 (υ $C_{(\alpha,\beta)}$ = O); 1597 (υ -HC_α=C_βH).

^{1}H NMR 500MHz $CDCl_3$ (δ ppm, m, J): 7.98 ppm (d, 8.3 Hz, H-2'/H-6'); 7.76 ppm (br. d, 15.6 Hz, H-β/H-7); 7.63 ppm (d, 8.2 Hz, H-3'/H-5'); 7.52 ppm (d, 8.8 Hz, H-2/H-6); 7.32 ppm (d, 15.6 Hz, H-α); 6.68 ppm (d, 8.6 Hz, H-3/H-5); 3.02 ppm (q, 7.0 Hz, H-9/H-10); 2.20 ppm (s, H-8).

^{13}C NMR 125MHz $CDCl_3$ (δ ppm): 25.0 ppm (C-8); 40.4 ppm (C-9;C-10); 119.4 ppm (C_α); 141.9 ppm(C_β); 112.1-152.9 ppm ($C_{aromatics}$); 168.3 ppm (C=O, amide); 189.5 ppm (C=O, α-β).

g) N-[4-[3-(4-Bromophenyl)-1-oxo-2-propen-1-yl]phenyl]acetamide (**ChBr**): $C_{17}H_{14}BrNO_2$; Reaction Yield 74 %; Molecular Weight: 344,21 $g.mol^{-1}$; CAS Number:

690672-56-1; ε_{DMSO} (333 nm): 34136 $mol^{-1}.L.cm^{-1}$; HRESI-MS: $[M+H]^+$ = m/z 344.0272

FTIR (cm^{-1}): 3306 (υ -N-H); 1678 (br, υ -NHC=O , $C_{(\alpha,\beta)}$ = O); 1612 (υ -HC_{α}=C_{β}H); 980 (υ C_{Ph}-Br).

^{1}H NMR 500MHz DMSO-d_6 (δ ppm, m, J): 10.32 ppm (s, H-7); 8.14 ppm (d, 8.7 Hz, H-2'/H-6'); 7.97 ppm (d, 15.8 Hz, H-β); 7.84 ppm (d, 8.5 Hz, H-3'/H-5'); 7.76 ppm (d, 8.6 Hz, H-3/H-5); 7.66 ppm (m, H-2/H-6/ H-α); 7.52 ppm (d, 8.8 Hz, H-2/H-6); 2.10 ppm (s, H-8).

^{13}C NMR 125MHz $CDCl_3$ (δ ppm): 24.2 ppm (C-8); 118.3 ppm (C_{α}); 143.8 ppm(C_{β}); 122.1-152.9 ppm ($C_{aromatics}$); 143.8 ppm (C=O, amide); 187.4 ppm (C=O, α-β).

h) N-[4-[(2E)-3-(4-Nitrophenyl)-1-oxo-2-propen-1-yl]phenyl]acetamide **(ChNO2)**: $C_{17}H_{14}N_2O_4$; Reaction Yield: 27 %; Molecular Weight: 310,31 $g.mol^{-1}$; CAS Number: 1015084-97-5; ε_{DMSO} (339 nm): 28055 $mol^{-1}.L.cm^{-1}$; HRESI-MS: $[M+H]^+$ = m/z 311.10221

FTIR (cm^{-1}): 3304 (υ -N-H); 1683 (υ -NHC=O); 1657 (υ $C_{(\alpha,\beta)}$ = O); 1600 (υ -HC_{α}=C_{β}H); 1513, 1345 (υ N-O).

^{1}H NMR 500MHz DMSO-d_6 (δ ppm, m, J): 10.36 ppm (s, H-7); 8.29 ppm (d, 8.8 Hz, H-3/H-5); 8.17 ppm (d, 8.7 Hz, H-2'/H-6'/H-3'/H-5'/H-β); 7.79 ppm (m, H-2/H-6/ H-α); 2.11 ppm (s, H-8).

^{13}C NMR 125MHz DMSO-d_6 (δ ppm): 24.2 ppm (C-8); 118.3 ppm (C_{α}); 144.1 ppm(C_{β}); 123-148.1 ppm ($C_{aromatics}$); 169.2 ppm (C=O, amide); 187.3 ppm (C=O, α-β).
